# FTY720 induces non-canonical phosphatidylserine externalization and cell death in acute myeloid leukemia

**DOI:** 10.1038/s41419-019-2080-5

**Published:** 2019-11-07

**Authors:** Megan M. Young, Van Bui, Chong Chen, Hong-Gang Wang

**Affiliations:** 1 Department of Pediatrics, Pennsylvania State University College of Medicine, Hershey, PA 17033 USA; 2 Department of Pharmacology, Pennsylvania State University College of Medicine, Hershey, PA 17033 USA

**Keywords:** Cancer therapy, Acute myeloid leukaemia, Cell death, Preclinical research

## Abstract

FTY720 (fingolimod) is a FDA-approved sphingosine analog that is phosphorylated in vivo to modulate sphingosine-1-phosphate receptor (S1PR) signaling for immunosuppression in patients with refractory multiple sclerosis. FTY720 also exhibits promising anticancer efficacy in several preclinical models. While FTY720-induced cytotoxicity is not due to S1PR signaling, the mechanism remains unclear and is reported to occur through various cell death pathways. Here, we performed a systematic, mechanistic study of FTY720-induced cell death in acute myeloid leukemia (AML). We found that FTY720 induced cell death in a panel of genetically diverse AML cell lines that was accompanied by rapid phosphatidylserine (PS) externalization. Importantly, FTY720-induced PS exposure was not due to any direct effects on plasma membrane integrity and was independent of canonical signaling by regulated cell death pathways known to activate lipid flip-flop, including caspase-dependent apoptosis/pyroptosis, necroptosis, ferroptosis, and reactive oxygen species-mediated cell death. Notably, PS exposure required cellular vacuolization induced by defects in endocytic trafficking and was suppressed by the inhibition of PP2A and shedding of Annexin V-positive subcellular particles. Collectively, our studies reveal a non-canonical pathway underlying PS externalization and cell death in AML to provide mechanistic insight into the antitumor properties of FTY720.

## Introduction

The asymmetric distribution of lipids across the inner and outer leaflets of the plasma membrane is essential for cellular integrity and signaling. Phosphatidylcholine (PC), sphingomyelin (SM), and glycosphingolipids are abundant in the exoplasmic leaflet, whereas phosphatidylserine (PS), phosphatidylethanolamine (PE), and phosphatidylinositol (PI) predominantly reside in the cytoplasmic leaflet^1,2^. Protein transporters maintain plasma membrane phospholipid asymmetry. Flippases (type IV P-type ATPases) and floppases transport substrate-specific lipids across the concentration gradient in an ATP-dependent manner, while scramblases mediate the non-selective and ATP-independent bidirectional transport of lipids down the concentration gradient^3^. The loss of asymmetry and exposure of PS on the cell surface has been used as a hallmark of apoptosis for several decades^4^; however, recent reports have shown that PS exposure also occurs during necroptosis and ferroptosis^5–9^. In addition, viable cells transiently externalize PS under several physiological conditions, including the activation of T lymphocytes^10,11^, B lymphocytes^12,13^, and mast cells^14,15^, sperm capacitation^16^ and on tumor cells and vascular endothelial cells within the tumor microenvironment^17,18^.

FTY720 (fingolimod; trade name Gilenya, Novartis) is a FDA-approved immunosuppressive agent for the treatment of refractory multiple sclerosis^19^. The sphingosine analog is phosphorylated in vivo by sphingosine kinase 2 to generate a metabolite that acts through cell surface sphingosine-1-phosphate (S1P) G protein-coupled receptors to sequester lymphocytes in the lymph nodes^20–24^. While the S1P receptor (S1PR)-mediated immunosuppressive effects occur with nanomolar affinity, micromolar concentrations of FTY720 display significant anticancer effects in a number of cancer models^25,26^, including acute myeloid leukemia (AML)^27–30^. Notably, cytotoxicity is not attributable to S1PR signaling and has been proposed to result from FTY720’s intracellular protein targets. FTY720 inhibits several enzymes in the sphingolipid pathway, including sphingosine kinase 1^21,31^, ceramide synthase^32^, and S1P lyase^33^. In addition, FTY720 activates protein phosphatase 2 A (PP2A) via direct association with the endogenous PP2A inhibitor I2PP2A/SET^34–36^. Despite exhibiting efficacy in many cancer models, an overarching mechanism of FTY720-induced cell killing has not been identified, but rather cell death is reported to occur through the reactivation of PP2A, induction of bioenergetic stress and/or initiation of canonical regulated cell death (RCD) pathways, including apoptosis, autophagy, and necroptosis^27–29,35–45^. The broad array of signaling molecules and simultaneous induction of multiple mechanisms of RCD reported for FTY720 led us to perform a systematic mechanistic analysis of FTY720-induced cell death in AML. Here, we found that FTY720 induced the externalization of PS through a non-canonical cell death pathway in AML cells that is mediated by endocytic dysfunction and cellular vacuolization.

## Materials and methods

### Cell culture

MV4-11 (ATCC, Manassas, VA, USA, #CRL-9591), THP1 (ATCC, #TIB-202) and MOLM13 (DSMZ, Braunschweig, Germany, #ACC 554) AML cell lines were maintained in Iscove’s Modified Dulbecco’s Medium (IMDM, for MV4-11 and THP1; Life Technologies, Carlsbad, CA, #12200069) or Roswell Park Memorial Institute 1640 (RPMI-1640, for MOLM13; VWR, Radnor, PA, USA, #45000-396) supplemented with 10% FBS (VWR, #97068-091), 100 μg/ml of streptomycin, 100 units/ml penicillin, and 250 ng/ml amphotericin B (VWR, #45000-616). All cell lines were routinely verified to free of *Mycoplasma* contamination using the MycoAlert Mycoplasma detection kit (Lonza, Basel, Switzerland, #LT07-318).

### Chemicals and reagents

FTY720 (dissolved in DMSO; #10006292), FTY720-phosphate (dissolved in DMSO; #10008639), NBD-FTY720 (dissolved in DMSO; #16841), calyculin A (#19246) and necrosulfonamide (#20844) were purchased from Cayman Chemical Company (Ann Arbor, MI, USA). FITC conjugated Annexin V (#640945), allophycocyanin (APC) conjugated Annexin V (#640941) and 7-aminoactinomycin D (7-AAD; #420404) were purchased from BioLegend (San Diego, CA, USA). Annexin V Alexa Fluor 594 conjugate (#A13203), YOYO-3 Iodide (#Y3606), CellTrace-carboxyfluorescein succinimidyl ester (CSFE) (#C34554) and CellEvent Caspase-3/7 Green Detection Reagent (#C10423) were purchased from Invitrogen (Thermo Fisher Scientific, Inc.; Waltham, MA, USA). (1S,3R)-RAS-selective lethal 3 (#SML2234), ferrostatin-1 (#SML0583), GSK’872 (#530389), methyl-β-cyclodextrin (#C4555), N-acetyl-L-cysteine (#A7250), necrostatin-1 (#N9037), Pitstop-2 (#SML1169) and DMSO (#D2438) were purchased from Sigma-Aldrich (St. Louis, MO, USA). The following chemicals were purchased from the indicated sources: carbobenzoxy-valyl-alanyl-aspartyl-[O-methyl]-fluoromethylketone (z-VAD-fmk; #HY-16658) from MedChemExpress (Monmouth Junction, NJ, USA), E64d (#S7393) from Selleck Chemicals (Houston, TX, USA), pepstatin A (#260-085) and dynasore (#270-502) from Enzo Life Sciences, Inc. (Farmingdale, NY, USA), and Bafilomycin A1 (#AAJ61835MCR) from Thermo Fisher Scientific.

### Antibodies

Unconjugated mouse anti-human CD98 (4F2hc, solute carrier family 3 member 2) Ab (#556074) and APC-conjugated goat anti-mouse Ig Ab (#550826) were purchased from BD BioSciences (San Jose, CA, USA). Mouse IgG1κ isotype control Ab (#400123-BL) was obtained from Biolegend (San Diego, CA, USA). Rabbit anti-human ATG7 Ab (#8558) was purchased from Cell Signaling Technology (Danvers, MA, USA), and mouse anti-β-actin Ab (#A5441) was from Sigma-Aldrich. IRDye 800CW donkey anti-rabbit (#925-32213) and IRDye 680RD donkey anti-mouse (#925-68072) secondary antibodies were purchased from LI-COR (Lincoln, NE, USA).

### Flow cytometry

300,000 cells were seeded at 0.4 × 10^6^ cells/ml and treated as described in the figure legends. To monitor PS externalization and cell death, cells were harvested, washed twice in ice-cold PBS and re-suspended in ice-cold Annexin V (Ann V) Binding Buffer (10 mM HEPES, pH 7.4, 140 mM NaCl, 2.5 mM CaCl_2_). 100,000 cells were incubated with FITC- or APC-Ann V (1:50 dilution) and 7-AAD (1:50 dilution) for 10 min at room temperature, protected from light, followed by analysis within 1 h. For detection of caspase-3/7 activity, cells were treated in the presence of 1 µM CellEvent Caspase-3/7 Green Detection Reagent prior to Ann V/7-AAD staining. Note that NSA displays high auto-fluorescence in the 488 nm laser and was excluded from analysis with this reagent. The staining of surface CD98 was adapted from Finicle et al.^46^. Briefly, cells were harvested and washed twice with ice-cold FACS blocking buffer (10% FBS, 0.05% sodium azide in PBS). 150,000 cells were incubated with human Fc Block on ice for 10 min according to the manufacturer’s protocol followed by the addition of unconjugated anti-CD98 Ab (1:100) or an equal concentration of IgG1κ isotype control Ab for 30 min on ice. Cells were washed twice with FACS wash buffer (2% FBS, 0.05% sodium azide in PBS) prior to the addition of APC-conjugated goat anti-mouse Ig secondary Ab and incubated on ice for 20 min, protected from light. Cells were washed twice with FACS wash buffer and re-suspended in Ann V Binding buffer containing FITC-Ann V (1:50 dilution) and 7-AAD (1:50 dilution) for 10 min prior to analysis by flow cytometry. For surface CD98 levels, the APC median fluorescence intensity for each treatment was normalized to cells treated with DMSO for 30 min and is presented as the percent relative to control. Flow cytometry was performed using a BD FACS Canto (10-color) instrument (BD Biosciences, San Jose, CA, USA) in the Penn State College of Medicine Flow Cytometry Core Facility. Data was analyzed using FlowJo software (Version 10.5.3, San Carlos, CA, USA).

### IncuCyte live-cell analysis

60,000 cells (MV4-11, MOLM13; cell density of 0.3 × 10^6^ cells/ml) or 50,000 cells (THP1; cell density of 0.25 × 10^6^ cells/ml) were seeded in a 96-well plate and treated as described in the figure legends. Where indicated, treatment medium contained Ann V Alexa Fluor 594 conjugate (Ann V-AF594; 1:400 dilution), FITC-Ann V (1:400 dilution) and/or the cell-impermeable, nucleic acid stain YOYO-3 (5 nM). Ann V binding to PS is calcium-dependent, and calcium concentrations in cell culture medium are variable. While IMDM contains 1.5 mM calcium, RPMI-1640 contains only 0.42 mM calcium; thus, for MOLM-13 live cell analysis, RPMI-1640 medium was supplemented with 1.1 mM calcium chloride. Fluorescence and phase images (four images per well, three wells per treatment) were obtained at the indicated intervals using the IncuCyte S3 Live-Cell Analysis System (Essen BioScience, Ann Arbor, MI, USA; 10× or 20× objectives). Images were quantified using the Basic Analyzer or Cell-by-Cell Analysis (Essen BioScience, #9600-0031) modules of the IncuCyte S3 Software (Essen BioScience, Version 2019A). For analysis with the Basic Analyzer, cells were labeled with CellTrace-CSFE prior to treatment in order to allow for accurate quantification of the total cell count per image. For cell labeling, cells were resuspended to a density of 2 × 10^6^ cells/ml in serum-free medium containing 0.2 μM CellTrace-CSFE and incubated for 5 min at 37 °C. To limit adverse toxicity by unbound CSFE, cells were washed two times in medium containing 10% FBS followed by resuspension in fresh serum-containing medium and incubation at 37 °C for 20 min with periodic inverting. Cell pellets were resuspended in fresh medium prior to seeding and treatment. For these experiments, CSFE-labeled cells were seeded in duplicate plates, treated in the presence of Ann V-AF594 or YOYO3 and imaged in parallel.

### CRISPR/Cas9 genome editing

Non-targeting (CR-NT) and ATG7 deficient (CR-ATG7) THP1 cell lines were previously described^47^. MV4-11 and MOLM13 cell lines were generated by CRISPR/Cas9 genome editing. Single guide RNA (sgRNA) targeting ATG7 (5′-AACTCCAATGTTAAGCGAGC-3′) or non-targeting control sgRNA (5′-GACCGGAACGATCTCGCGTA-3′) were subcloned into pLenti-CRISPR-V2 (Addgene, #52961). Lentiviral packaging was performed in HEK293 cells, as previously described^48^, and transduced AML cell lines were selected for 5 days in puromycin. Single clones were isolated and screened for gene disruption by western blotting and successfully targeted clones were pooled. For western blot analysis of the resultant cell lines, cell pellets were lysed in NP-40 lysis buffer (150 mM NaCl, 50 mM Tris-HCl pH 8.0, 1% NP-40) containing protease inhibitor cocktail (Sigma-Aldrich, #P8340), cleared by centrifugation and assayed for protein concentration by BCA protein assay (Thermo Scientific, #23225). Equal amounts of total protein were loaded per well, separated by SDS-PAGE, and transferred to Immobilon PVDF membrane (MilliporeSigma, #ISEQ00010). Membranes were blocked using Odyssey Blocking Buffer (LI-COR, #927-50003) and probed with the indicated primary and secondary antibodies: ATG7 (1:1,000 dilution), β-actin (1:20,000 dilution), IRDye 800CW-conjugated donkey anti-rabbit Ab (1:15,000 dilution) and IRDye 680RD-conjugated donkey anti-mouse Ab (1:15,000 dilution). Blots were imaged using the Odyssey CLx Imaging System and Image Studio Software (LI-COR).

### Statistical analyses and reproducibility

Statistical analysis was performed using GraphPad Prism software (Version 8.1.1, GraphPad, San Diego, CA, USA). Threshold for significance for each test was set at 95% confidence (*p* ≤ 0.05). Statistical tests, post-hoc analyses, and statistical significance are indicated in each figure legend. All data are representative of at least three independent experiments with the number of technical replicates indicated in each figure legend. Data are presented as the mean ± standard deviation, and no data was excluded from analysis.

## Results

### FTY720 induces the exposure of PS on the surface of AML cells prior to cell death and in the absence of its phosphorylation

**Fig. 1:**
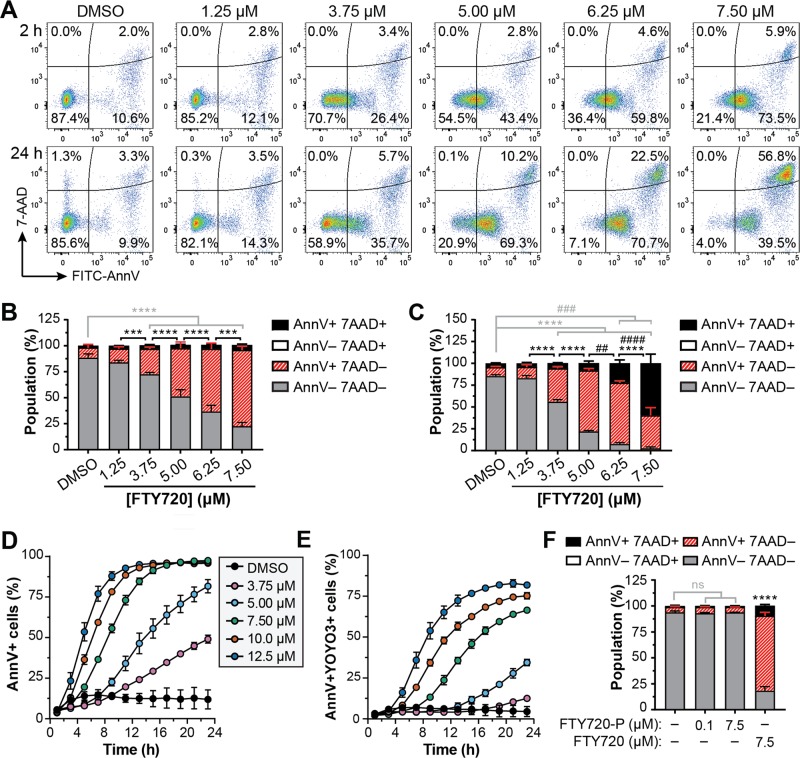
FTY720 induces the exposure of PS on the surface of AML cells prior to cell death and in the absence of its phosphorylation. **a**–**c** MV4-11 cells were treated with DMSO vehicle control or the indicated doses of FTY720 for 2 h (top panel of **a**; **b**) or 24 h (bottom panel of **a**; **c**), stained with FITC-Annexin V (FITC-Ann V) and 7-AAD and analyzed by flow cytometry. **a** Representative flow cytometry plots are shown. **b**, **c** Populations are presented as the mean ± SD, *n* = 3. Statistical significance for AnnV + 7AAD− (*) and AnnV + 7AAD+ (#) populations was determined by two-way ANOVA followed by Tukey’s multiple comparison test. ****p ≤ 0.0001; ***p ≤ 0.001; ^####^*p* ≤ 0.0001; ^###^*p* ≤ 0.001; ^##^*p* ≤ 0.01. **d**, **e** MV4-11 cells were treated with the indicated doses of FTY720 in the presence of FITC-Ann V and YOYO3, imaged with the IncuCyte Live-Cell Analysis System, and quantified using the Cell-by-Cell Analysis module. Mean ± SD, *n* = 3. Note: error bars are shown for all data points but may be masked by symbols. **d** Percent of FITC-Ann V positive cells versus time. **e** Percent of FITC-Ann V and YOYO3 double-positive cells versus time. **f** MV4-11 cells were treated with the indicated doses of FTY720-phosphate (FTY720-P) or FTY720 for 4 h, stained with APC-Ann V and 7-AAD and analyzed by flow cytometry. Mean ± SD, *n* = 3. Statistical significance for the AnnV + 7AAD− population was determined by two-way ANOVA followed by Tukey’s multiple comparison test. ns, not significant; ****p ≤ 0.0001

To examine AML cell death in response to FTY720, MV4-11 cells were treated with a dose response of FTY720 and plasma membrane asymmetry and integrity were examined by staining with fluorophore-conjugated Annexin V (Ann V) and the cell-impermeable nucleic acid binding dye 7-AAD. Unexpectedly, FTY720 induced a dose-dependent increase in Ann V binding after only 2 h (Fig. 1a, top panel and 1b). Notably, this response occurred in the absence of 7-AAD staining and at doses significantly below the reported critical micellar concentration of 75 μM for FTY720 in aqueous medium^49^ to indicate that the sphingosine analog was not having a direct effect on plasma membrane integrity or causing membrane dissolution. The exposure of PS by FTY720 did not always immediately precede cell death, as a significant population of Ann V-positive cells remained viable after 24 h of treatment with 3.75 μM or 5 μM FTY720 (Fig. 1a–c). Moreover, PS exposure on viable cells was 1-2 orders of magnitude below that of apoptotic cells (Fig. S1a). In contrast, significant cytotoxicity was induced after 24 h treatment with FTY720 at concentrations above 5 μM, at which point the cells became 7-AAD positive and demonstrated enhanced FITC-Ann V binding (Fig. 1a, c; Fig. S1a).

To analyze the cell death kinetics in real-time, MV4-11 cells were treated in the presence of FITC-Ann V and the cell-impermeable nucleic acid stain YOYO3 and imaged using the IncuCyte Live Cell Analysis System. Similar to flow cytometry, FTY720 induced a rapid increase in Ann V-positive cells within several hours of treatment and led to dose-dependent cell death (Fig. 1d–e). Despite a similar phenotype, the kinetics of Ann V binding was notably delayed during live cell imaging compared to flow cytometry analysis. This is likely attributable to differences in the staining conditions and/or fluorescence detection sensitivity between the two methods. Similar phenotypes were also observed in THP1 (Fig. S1b, c) and MOLM13 (Fig. S1d, e) cells to demonstrate that FTY720 induces robust Ann V positivity but dose-dependent cell death across genetically diverse AML cell lines. While the loss of membrane asymmetry precedes the commitment to cell death, Ann V positive cells can resume cell growth upon the removal of the death signal and restoration of phospholipid asymmetry^50^. Similarly, removal of FTY720 after 4 h (at which time PS externalization is significantly induced) impaired FITC-Ann V binding and cell death compared to cells cultured in the continued presence of the FTY720 (Fig. S1f, g). Furthermore, consistent with previous reports demonstrating that FTY720-induced cytotoxicity does not occur due to its phosphorylation^36–38,51^, FTY720-phosphate failed to induce Ann V positivity or cell death at equimolar dosing to FTY720 or at a nanomolar concentration that is sufficient for S1PR-mediated effects^20,52–54^ (Fig. 1f, Fig. S1h). Taken together, these results demonstrate that FTY720-induced cell death in AML cell lines is accompanied by the rapid loss of plasma membrane asymmetry in the absence of its phosphorylation.

### The exposure of PS and cytotoxicity induced by FTY720 is independent of caspase activation and necroptosis

**Fig. 2:**
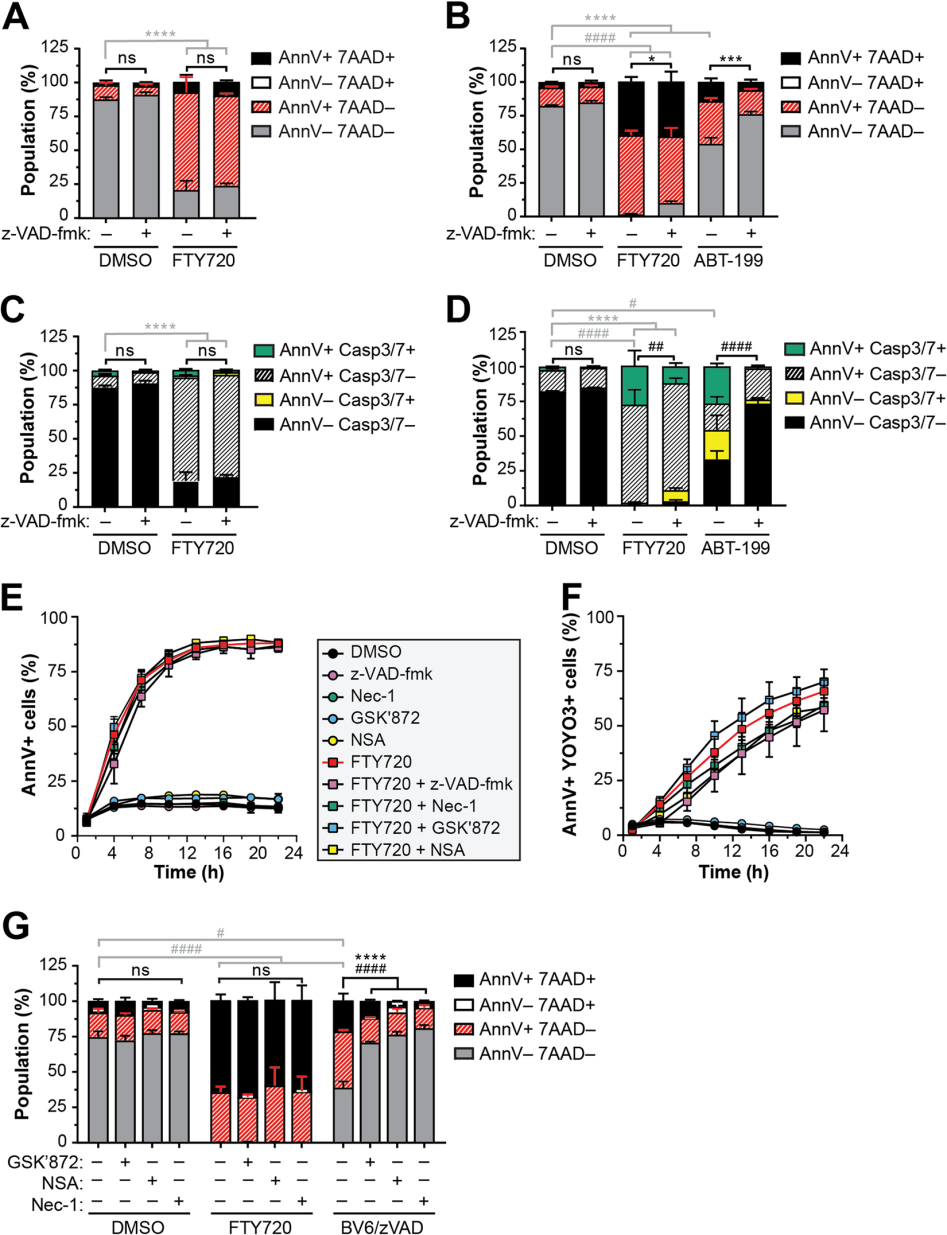
The exposure of PS and cytotoxicity induced by FTY720 is independent of caspase activation and necroptosis. **a–d** MV4-11 cells were seeded in medium containing CellEvent Caspase-3/7 Green and treated with 7.5 μM FTY720 or 250 nM ABT-199 (positive control) in the presence or absence of 50 μM z-VAD-fmk for 4 h (**a**, **c**) or 24 h (**b**, **d**), stained with APC-Ann V and 7AAD and analyzed by flow cytometry. Mean ± SD, *n* = 3. For **a** and **b**, statistical significance for AnnV + 7AAD− (*) and AnnV + 7AAD + (#) populations was determined by two-way ANOVA followed by Tukey’s multiple comparison test. ns, not significant; ****p ≤ 0.0001; ***p ≤ 0.001; *p ≤ 0.05; ^####^*p* ≤ 0.0001. For **c** and **d**, statistical significance for AnnV + Casp3/7− (*) and AnnV + Casp3/7 + (#) populations was determined by two-way ANOVA followed by Tukey’s multiple comparison test.: ns, not significant; ****p ≤ 0.0001; ^####^*p* ≤ 0.0001; ^##^*p* ≤ 0.01; ^#^*p* ≤ 0.05. **e**, **f** MV4-11 cells were treated with DMSO or 7.5 μM FTY720 in the presence or absence of 50 μM z-VAD-fmk, 30 µM Necrostatin-1 (Nec-1), 15 µM GSK’872 or 1 µM necrosulfonamide (NSA) in medium containing FITC-Ann V and YOYO3. Cells were imaged using the IncuCyte Live-Cell Analysis System and quantified with the Cell-by-Cell Analysis module. Mean ± SD, *n* = 3. Note: error bars are shown for all data points but may be masked by symbols. **e** Percent of FITC-Ann V positive cells versus time. **f** Percent of FITC-Ann V and YOYO3 double-positive cells versus time. **g** MV4-11 cells were treated with 7.5 μM FTY720 or 600 nM BV6 plus 20 μM z-VAD-fmk (BV6/zVAD; positive control) in the presence or absence of 15 µM GSK’872, 1 µM NSA or 30 µM Nec-1 for 24 h and stained with APC-Ann V and 7-AAD for flow cytometry analysis. Mean ± SD, *n* = 3. Statistical significance for AnnV + 7AAD− (*) and AnnV + 7AAD+ (#) populations was determined by two-way ANOVA followed by Tukey’s multiple comparison test. ns, not significant; ****p ≤ 0.0001; ^####^*p* ≤ 0.0001; ^#^*p* ≤ 0.05

The high binding affinity of recombinant Ann V for PS has been used as a marker of apoptotic cells for several decades^4^. Exposure of PS on the exofacial leaflet of the plasma membrane during early apoptosis results from the caspase-dependent inactivation of the phospholipid flippase ATP11C and caspase-mediated activation of the phospholipid scramblase Xkr8^55,56^. PS flipping to the outer leaflet also occurs downstream of inflammatory caspase activation during gasdermin D-mediated pyroptosis by the calcium-mediated activation of phospholipid scramblase TMEM16F (also known as anoctamin 6/ANO6)^57^. To examine the role of caspase activation in FTY720-induced PS exposure and cytotoxicity, MV4-11 cells were treated in the presence of the pan-caspase inhibitor z-VAD-fmk and a fluorogenic caspase-3/7 detection probe (Fig. 2a–d). For these and subsequent mechanistic studies, a single dose of FTY720 was selected at which approximately 50% cell death was observed after 24 h. While z-VAD-fmk significantly suppressed PS exposure and caspase-3/7 activation in response to the apoptosis inducer ABT-199 (Fig. 2b, d), the inhibitor failed to block FTY720-induced PS exposure (Fig. 2a and e) or subsequent cell death (Fig. 2b, f). Moreover, the Ann V-positive cells induced by FTY720 were negative for the fluorgenic caspase-3/7 detection probe (Fig. 2c). Interestingly, a subpopulation of cells treated for 24 h with FTY720 were positive for both caspase-3/7 activity and Ann V binding (green bars in Fig. 2d). While this population was significantly suppressed by z-VAD-fmk (Fig. 2d), the inhibitor failed to dramatically rescue Ann V binding or cell death (Fig. 2b, e, f) to suggest that caspase activation in these cells may occur as a consequence rather than initiator of cellular demise. Similarly, z-VAD-fmk failed to block the externalization of PS or cell death induced by FTY720 in THP1 (Fig. S2a, b) and MOLM13 cell lines (Fig. S2c).

Contrary to traditional dogma, the current literature demonstrates that the loss of plasma membrane asymmetry is not restricted to caspase-dependent RCD. For example, necroptotic cells externalize PS after the translocation of phosphorylated mixed-lineage kinase domain-like pseudokinase (MLKL) to the plasma membrane and prior to the loss of plasma membrane integrity^5–7^. As a recent study has implicated a complex between ceramide and receptor-interacting protein kinase 1 (RIPK1) in the induction of type II necroptosis by FTY720 in human lung cancer cell lines^37^, we hypothesized that PS exposure in our system may be occurring by necroptosis. To test our hypothesis, we utilized three inhibitors of the necroptotic pathway: necrostatin-1 (Nec-1; RIPK1 inhibitor), GSK’872 (receptor-interacting protein kinase 3, RIPK3 inhibitor) and necrosulfonamide (NSA; MLKL inhibitor). Consistent with previous reports^5–7^, induction of necroptosis in MV4-11 cells by the SMAC (second mitochondria-derived activator of caspases) mimetic BV6 plus z-VAD-fmk (BV6/zVAD) resulted in cell death accompanied by Ann V-positive, 7-AAD-negative cells that was significantly rescued by inhibitors of RIPK1, RIPK3, and MLKL (Fig. 2g, Fig. S2d). Importantly, all three necroptosis inhibitors failed to alter Ann V binding or cell death in FTY720-treated MV4-11 cells (Fig. 2e–g). Furthermore, no increase in caspase-3/7 activity was detected in cells treated with FTY720 in the presence of Nec-1 or GSK’872 to indicate that cell death under these conditions was not a result of the crosstalk between apoptosis and necroptosis (Fig. S2d). Similarly, inhibitors of necroptosis had no effect on FTY720-treated THP1 (Fig. S2a, b, e) and MOLM13 cells (Fig. S2c). Collectively, these results indicate that FTY720-induced externalization of PS and cell death in AML is independent of caspase-dependent apoptosis/pyroptosis and RIPK1/3-mediated necroptosis.

### Ferroptosis, ROS and autophagy are not responsible for the loss of plasma membrane asymmetry and integrity induced by FTY720

**Fig. 3:**
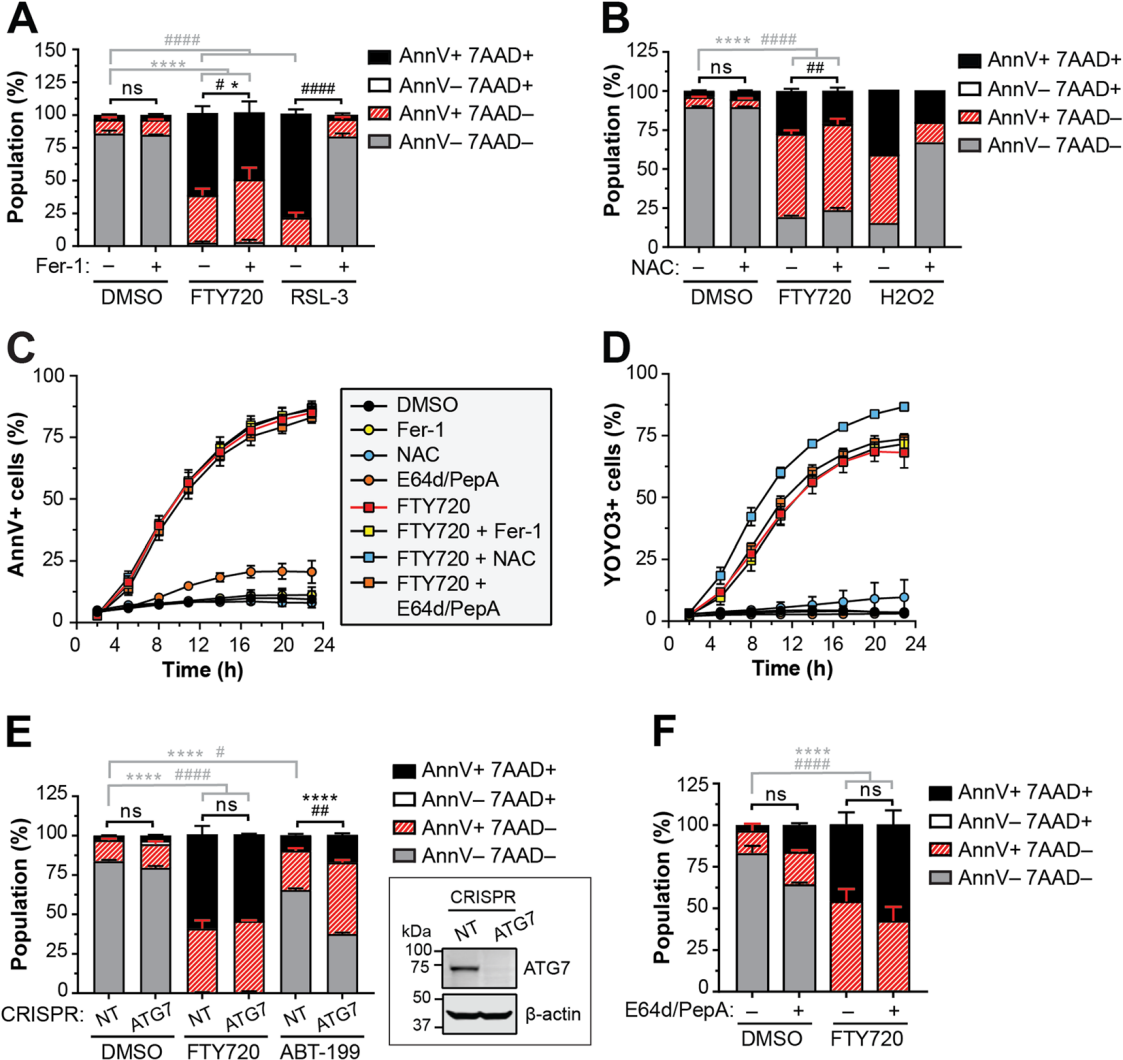
Ferroptosis, ROS and autophagy are not responsible for the loss of plasma membrane asymmetry and integrity induced by FTY720. **a** MV4-11 cells were treated with 7.5 µM FTY720 or 1 μM RSL-3 (positive control) in the presence or absence of 2 µM Ferrostatin-1 (Fer-1) for 24 h, stained with APC-Ann V and 7-AAD and analyzed by flow cytometry. Mean ± SD, *n* = 3. Statistical significance for AnnV + 7AAD− (*) and AnnV + 7AAD+ (#) populations was determined by two-way ANOVA followed by Tukey’s multiple comparison test. ****p ≤ 0.0001; *p ≤ 0.05; ^####^*p* ≤ 0.0001; ^#^*p* ≤ 0.05. **b** MV4-11 cells were treated with 7.5 µM FTY720 or 1.25 mM hydrogen peroxide (H_2_O_2_; positive control) in the presence or absence of 5 mM N-acetyl-cysteine (NAC) for 22 h, stained with APC-Ann V and 7-AAD and analyzed by flow cytometry. Mean ± SD, *n* = 3 (DMSO, FTY720); *n* = 1 (H_2_O_2_). Statistical significance for AnnV + 7AAD− (*) and AnnV + 7AAD+ (#) populations was determined by two-way ANOVA followed by Tukey’s multiple comparison test; ****p ≤ 0.0001; ^####^*p* ≤ 0.0001; ^##^*p* ≤ 0.01. **c**–**d** CSFE-labeled MV4-11 cells were treated with 7.5 μM FTY720 in the presence or absence of 2 µM Fer-1, 5 mM NAC or E64d/PepA (10 µg/mL each) in medium containing Ann V-AF594 (**c**) or YOYO3 (**d**). Images were obtained using the IncuCyte Live Cell Analysis System and quantified with the Basic Analyzer module. Mean ± SD, *n* = 3. Note: error bars are included for all data points but may be masked by symbols. **c** Percent of Ann V-AF594 positive CSFE-labeled cells versus time. **d** Percent of YOYO3 positive CSFE-labeled cells versus time. **e** CR-NT or ATG7-deficient MV4-11 cells were treated with 7.5 µM FTY720 or 250 nM ABT-199 (positive control) for 24 h, stained with APC-Ann V and 7-AAD and analyzed by flow cytometry. Mean ± SD, *n* = 3. Statistical significance for AnnV + 7AAD− (*) and AnnV + 7AAD+ (#) populations was determined by two-way ANOVA followed by Tukey’s multiple comparison test. ns, not significant; ****p ≤ 0.0001; ^####^*p* ≤ 0.0001; ^##^*p* ≤ 0.01; ^#^*p* ≤ 0.05. Immunoblot included in inset. **f** MV4-11 cells were treated with 7.5 µM FTY720 in the presence or absence of E64d/PepA for 24 h and subjected to Ann V/7AAD flow cytometric analysis. Mean ± SD, *n* = 3. Statistical significance for AnnV + 7AAD− (*) and AnnV + 7AAD+ (#) populations was determined by two-way ANOVA followed by Tukey’s multiple comparison test. ns, not significant; ****p ≤ 0.0001; ^####^*p* ≤ 0.0001

Peroxidation of plasma membrane lipids during ferroptosis or in response to ROS activates TMEM16F to induce phospholipid scrambling and the surface exposure of PS^7–9^. To examine the role of ferroptosis and ROS in FTY720-induced PS flipping and cell death, we utilized the ferroptosis inhibitor ferrostatin-1 (Fer-1) and ROS scavenger N-acetyl-cysteine (NAC), respectively. As expected, Fer-1 significantly rescued MV4-11 cell death in response to the glutathione peroxidase 4 (GPX4) inhibitor (1S,3R)-RAS-selective lethal 3 (RSL-3) (Fig. 3a), and NAC successfully blocked hydrogen peroxide-induced cell death (Fig. 3b). While Fer-1 and NAC induced a statistically significant decrease in FTY720-induced cell death, the small degree of rescue suggests that neither pathway is the primary initiator of FTY720-induced cell death (Fig. 3a, b). Furthermore, Fer-1 and NAC failed to inhibit the rapid Ann V positivity induced by FTY720 prior to cell death (Fig. 3c, d) to indicate that lipid peroxidation and ROS are not responsible for PS externalization. Similar results were obtained using THP1 (Fig. S3a–c) and MOLM13 cell lines (Fig. S3d).

FTY720-induced cell death has also been reported to occur in an autophagy-dependent manner^40,41^. To examine the role of autophagy upon FTY720 treatment of AML cells, we performed CRISPR/Cas9 genome editing of the essential autophagy gene ATG7 and pooled several successfully targeted single clones to generate autophagy-deficient AML cell lines (inset of Fig. 3e, Fig. S3e, f). While loss of ATG7 significantly sensitized MV4-11 cells to apoptosis induced by ABT-199 compared to non-targeting sgRNA control (NT), Ann V binding and cell death induced by FTY720 was not significantly altered (Fig. 3e, Fig. S3g, h). As an alternative approach, we inhibited autophagic degradation by treating cells with the lysosomal protease inhibitors E64d and pepstatin A. Consistently, inhibition of lysosomal degradation failed to suppress FTY720-induced cell death in MV4-11 (Fig. 3c, d and f), THP1 (Fig. S3a, b) and MOLM13 (Fig. S3d) cell lines. These results indicate that FTY720-induced cell death in AML is partially aided by oxidative stress but exposure of PS is independent of ROS, autophagy, and lysosomal protease activity.

### FTY720-induced PS externalization on the cell surface follows cellular vacuolization and occurs in a cholesterol- and energy-dependent manner

**Fig. 4:**
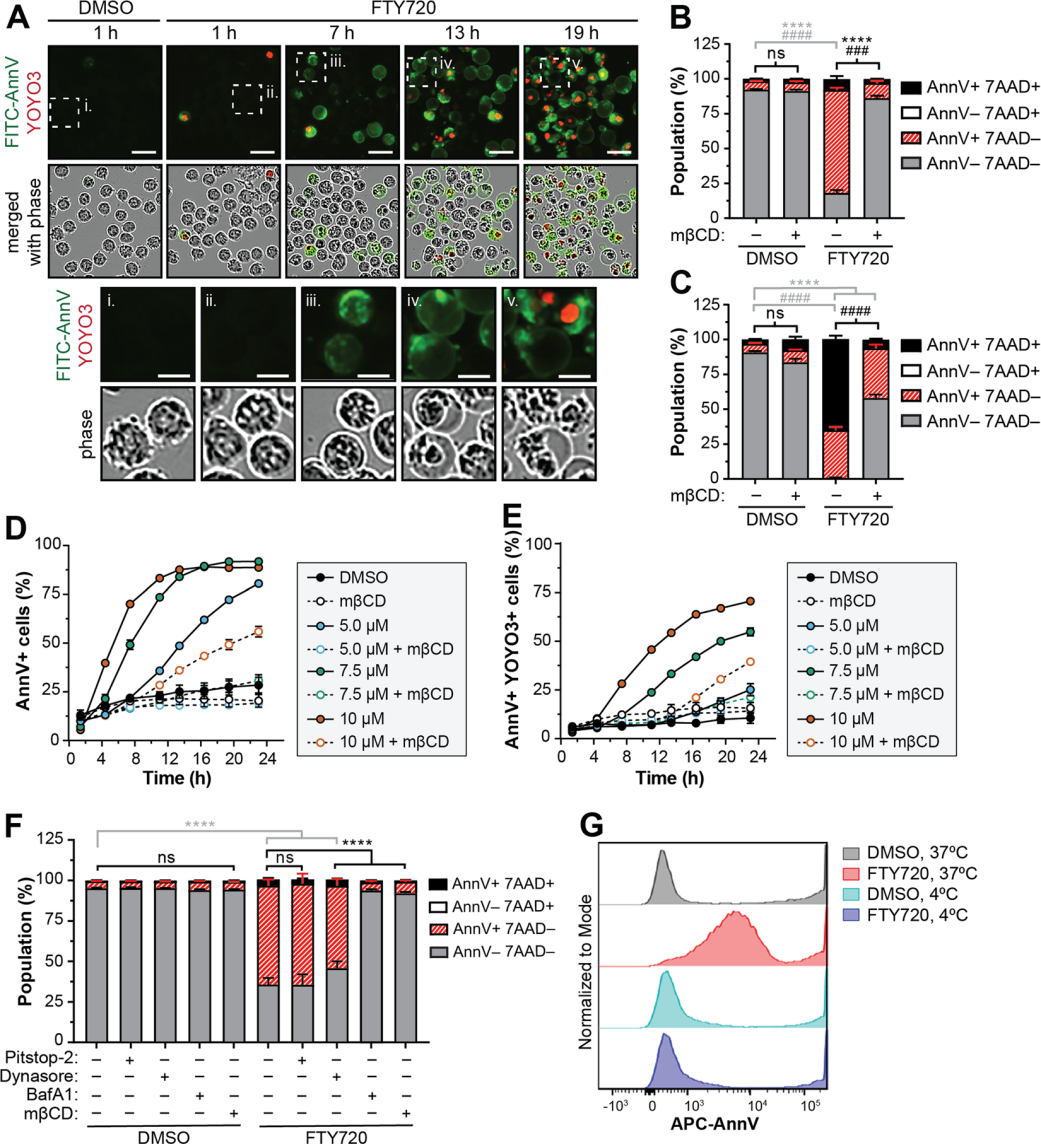
FTY720-induced PS externalization on the cell surface follows cellular vacuolization and occurs in a cholesterol- and energy-dependent manner. **a** MV4-11 cells were treated with DMSO or 7.5 μM FTY720 in the presence of FITC-Ann V and YOYO3 and imaged using the IncuCyte Live-Cell Analysis System. Images are representative of triplicate wells and three independent experiments. Scale bars represent 25 μm or 10 μm (enlarged, i–v). **b**, **c** MV4-11 cells were treated with DMSO or 7.5 µM FTY720 in the presence or absence of 2 mM methyl-β-cyclodextrin (mβCD) for 4 h (**b**) or 24 h (**c**), stained with APC-Ann V and 7-AAD and analyzed by flow cytometry. Mean ± SD, *n* = 3. Statistical significance for AnnV + 7AAD− (*) and AnnV + 7AAD+ (#) populations was determined by two-way ANOVA followed by Tukey’s multiple comparison test. ns, not significant; ****p ≤ 0.0001; ^####^*p* ≤ 0.0001; ^###^*p* ≤ 0.001. **d**, **e** MV4-11 cells were co-treated with the indicated doses of FTY720 and 2 mM mβCD in the presence of FITC-Ann V and YOYO3 and analyzed using the IncuCyte Live Cell Analysis System and Cell-by-Cell Analysis Module. Mean ± SD, *n* = 3. Note: error bars are shown for all data points but may be masked by symbols. **d** Percent of FITC-Ann V positive cells versus time. **e** Percent of FITC-Ann V and YOYO3 double-positive cells versus time. **f** MV4-11 cells were pre-treated with 30 µM Pitstop-2, 10 µM Dynasore, 100 nM BafA1, or 2 mM mβCD for 30 min followed by co-treatment with DMSO or 7.5 µM FTY720 for 3 h. Cells were stained with APC-Ann V and 7-AAD and analyzed by flow cytometry. Mean ± SD, *n* = 3. Statistical significance for the AnnV + 7AAD− (*) and AnnV + 7AAD+ (#) populations was determined by two-way ANOVA followed by Tukey’s multiple comparison test. ns, not significant; ****p ≤ 0.0001. **g** MV4-11 cells were treated with DMSO or 7.5 µM FTY720 at 37 °C or 4 °C (on ice) for 2 h, stained with APC-Ann V and 7-AAD and analyzed by flow cytometry. Representative histograms are shown

To gain more insight into a potential mechanism of PS exposure and cell death, we examined the cellular morphology and localization of FITC-Ann V in FTY720-treated cells. Previous work by us and others have shown that sphingolipid analogs induce cellular vacuolization due to defects in endocytic trafficking and that vacuolization is critical for cell death^58–62^. Interestingly, we observed that FTY720-induced vacuolization in MV4-11 cells (Fig. 4a, i–ii) occurred prior to the appearance of FITC-Ann V foci on the plasma membrane of viable cells (Fig. 4a, iii). The FITC-Ann V foci gradually assembled into clusters on the cell surface (Fig. 4a, iii), and after ~12–14 h, a faint FITC-Ann V signal surrounded the plasma membrane of cells with a dilated cytoplasm (Fig. 4a, iv). Ultimately, the dilated cellular structures collapsed and became positive for YOYO3 (Fig. 4a, v). To examine the localization of FTY720 in relation to Ann V foci on the plasma membrane, we attempted to utilize the fluorescently labeled analog, NBD-FTY720. Surprisingly, NBD-FTY720 failed to mimic FTY720-induced PS exposure, as Ann V binding was dramatically reduced and no longer dose dependent (Fig. S4a). As a result, we were unable to determine whether FTY720 co-localizes with Ann V-positive foci and advise caution in the use of fluorescent sphingolipid analogs during mechanistic studies.

To determine whether vacuolization is required for PS exposure, we blocked FTY720-induced vacuolization by co-treating cells with the cholesterol-depleting agent methyl-β-cyclodextrin (mβCD)^58,59^. Treatment with mβCD significantly impaired FTY720-induced Ann V binding and protected MV4-11 and THP1 cells from cell death (Fig. 4b, c, Fig. S4b, c). Moreover, this phenotype was confirmed by IncuCyte live cell imaging of MV4-11 or MOLM13 cells treated with several doses of FTY720 in the presence or absence of mβCD (Fig. 4d, e, Fig. S4d). As an alternative approach, endocytosis and vacuolization were impaired using the v-ATPase inhibitor Bafilomycin A1 (BafA1)^58,59,63^ or by incubating cells on ice. BafA1 significantly blocked FTY720-induced PS exposure (Fig. 4f), and importantly, this effect was independent of impaired lysosomal function as E64d and pepstatin A failed to alter Ann V binding (Fig. 3c). Similarly, FTY720-induced PS exposure was completely suppressed when cells were incubated at 4 °C (Fig. 4g) to indicate that PS exposure occurs through an energy-dependent process. As mβCD has been shown to inhibit clathrin-coated pits^64,65^, cells were also treated in the presence of the cell permeable clathrin inhibitor Pitstop-2 or dynamin GTPase inhibitor dynasore. While dynasore had a minor but significant effect in suppressing FTY720-induced PS exposure, Pitstop-2 had no effect to suggest that FTY720-induced vacuolization and PS exposure occur in a clathrin-independent manner (Fig. 4f). Interestingly, decreased plasma membrane cholesterol is a reported off-target effect of dynasore^66^, thus potentially offering an explanation for the small degree of rescue by the inhibitor. Taken together, FTY720-induced PS exposure occurs downstream of cholesterol-dependent endocytosis and cellular vacuolization.

### FTY720-induced PS exposure occurs after CD98 internalization and is suppressed by calyculin A-induced membrane shedding

**Fig. 5:**
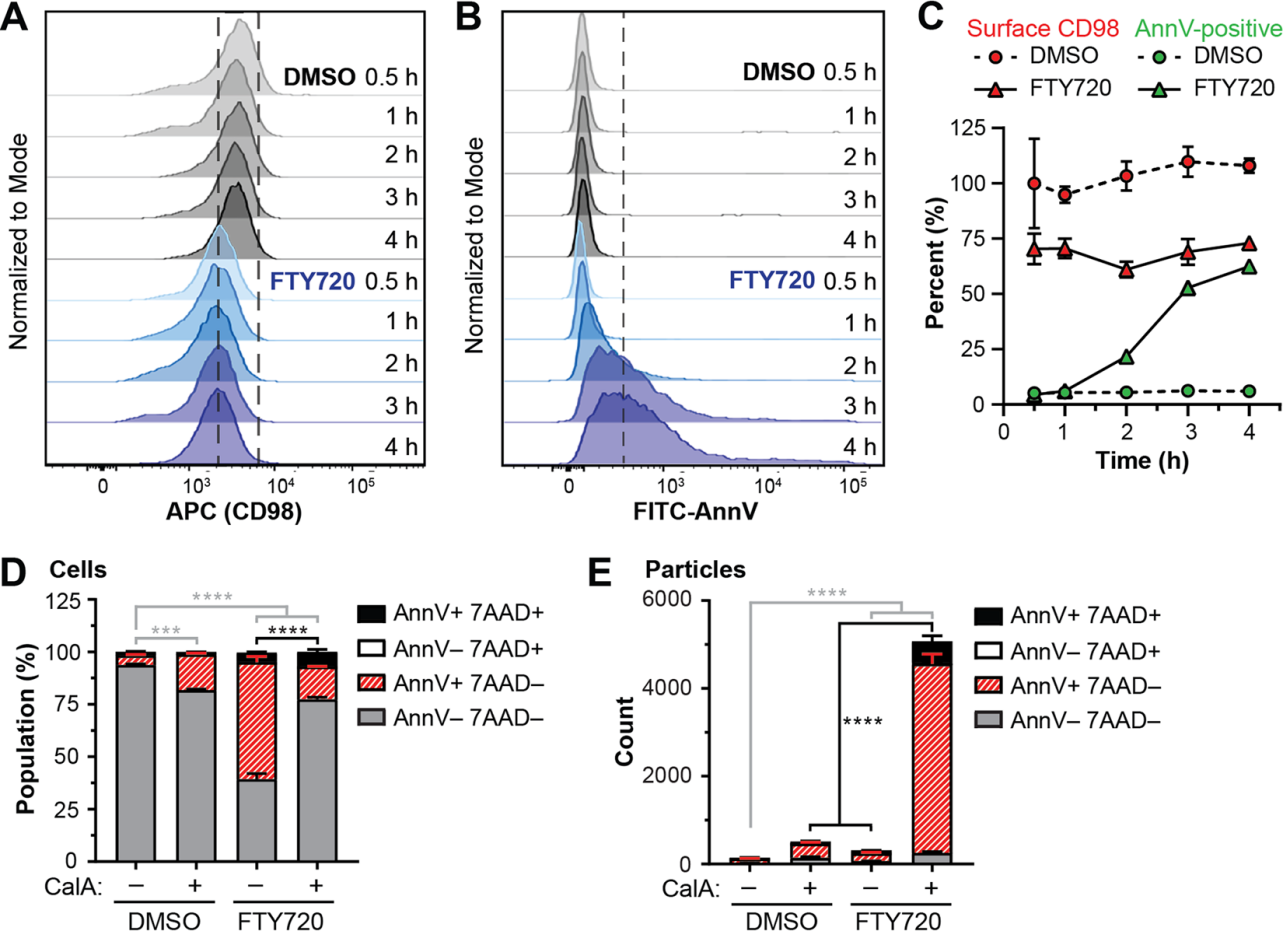
FTY720-induced PS exposure occurs after CD98 internalization and is suppressed by calyculin A. **a**, **b** MV4-11 cells were treated with DMSO or 7.5 μM FTY720 for the indicated time points and stained for surface CD98 (APC), FITC-Ann V and 7-AAD. Representative flow cytometry histograms are shown for viable cells. **a** APC (CD98). Dashed box is included as a reference for surface CD98 staining in DMSO treated cells. **b** FITC-Ann V. Dashed line reflects FITC-Ann V gating. **c** Data from (**a**) and (**b**) were quantified as described in the methods and presented as percent relative to control (for surface CD98) or as percent of total (Ann V). Mean ± SD, *n* = 3. **d**, **e** MV4-11 cells were pre-treated with DMSO or 5 nM calyculin A (CalA) for 30 min followed by co-treatment with vehicle or 7.5 μM FTY720 for 4 h. Cells were stained with APC-Ann V/7-AAD and analyzed by flow cytometry. Mean ± SD, *n* = 3. Statistical significance for the AnnV + 7AAD− (*) and AnnV + 7AAD+ (#) populations was determined by two-way ANOVA followed by Tukey’s multiple comparison test. ns, not significant; ****p ≤ 0.0001; ***p ≤ 0.001. **e** Cells were gated using forward scatter and side scatter and are presented as population of total cells. **e** Subcellular sized particles were gated using forward scatter and side scatter and are presented as number of particles

FTY720 and sphingolipid analogs downregulate lipid-raft associated nutrient transporters leading to bioenergetic stress and cell death^38,46,61,62,67^. Similar to PS externalization, nutrient transporter localization to the plasma membrane is restored upon cholesterol sequestration or drug removal^38,46^; thus, we wanted to examine the relationship between PS externalization and nutrient transporter internalization. Transient asymmetry in the surface area of the plasma membrane upon the addition of exogenous lipid can stimulate protein-aided flipping across the bilayer and the formation of endocytic vesicles^68^. While such a flippase for FTY720 is unknown, we hypothesized that the addition of FTY720 may alter PS distribution to stimulate the endocytosis of nutrient transporters. To test this hypothesis, we monitored the surface expression of the amino acid transporter chaperone CD98 (4F2hc/SLC3A2), which can be quantified by flow cytometry (Fig. S5a) and is internalized in response to FTY720^46^. Notably, surface CD98 levels rapidly decreased versus control by 30 min and significantly preceded FITC-Ann V binding (Fig. 5a–c) to indicate that PS flipping by FTY720 is not driving the endocytosis of nutrient transporters. Moreover, there was no direct correlation between surface CD98 levels and Ann V binding (data not shown).

Impaired nutrient transporter recycling in response to FTY720 and sphingolipid analogs is reported to occur through the PP2A-dependent inhibition of Arf6^46^. While inhibition of PP2A by calyculin A restored CD98 trafficking to the cell surface in FTY720-treated cells, Arf6 was inhibited in the presence of calyculin A even in the absence of FTY720^46^, leading us to hypothesize that another factor was restoring endocytic recycling in the presence of calyculin A. Co-treatment with calyculin A significantly rescued cells from FTY720-induced PS exposure (Fig. 5d, Fig. S5b, c). Interestingly, this was accompanied by a significant increase in Ann V-positive subcellular sized particles detected by flow cytometry (Fig. 5e, Fig. S5b, d). As the majority of Ann V-positive particles were negative for 7-AAD, we could eliminate the presence of apoptotic bodies. Unfortunately, rescue of cell death under these conditions could not be determined due to the toxicity of calyculin A alone upon prolonged incubation (data not shown). Collectively, these results suggest that inhibition of PP2A promotes plasma membrane shedding to restore plasma membrane asymmetry and vesicle trafficking.

## Discussion

Here, we show that FTY720 induces a non-canonical pathway underlying the exposure of PS and cell death in AML cells. While this study focused on the effects of FTY720 in AML cells, previous studies by other groups have investigated the effects of the sphingosine mimetic on adherent solid tumor cells. The sphingosine analog SKI-I induces a similar vacuolization-dependent cell death in human colon cancer cells^60^. Interestingly, SKI-I induces PS externalization in the absence of caspase activation to suggest that non-canonical PS exposure may also occur in solid tumor cells. In addition, FTY720-induced apoptosis has been reported in a number of solid tumor cells^40,41,69^; however, the contribution of non-canonical PS flipping to Ann V positivity in these models requires further investigation. Moreover, FTY720-induced cell death in human lung cancer cell lines is accompanied by a dramatic remodeling of the plasma membrane leading to blebbing and type II necroptosis^36,37^; however, a role for PS exposure was not determined. Further study of FTY720 is warranted in order to determine if non-canonical PS exposure is affected by cell adherence or is cell type dependent. Collectively, our results underscore the recent literature to emphasize that it is no longer appropriate to interpret Ann V binding as apoptotic cell death and that mechanistic determination of cell death requires the use of pharmacological and/or genetic inhibitors alongside appropriate controls. Surprisingly, FTY720-induced PS exposure occurred within 2 h and did not always precede cell death, as cells treated at sublethal doses maintained viability despite Ann V labeling one to two orders of magnitude below that of apoptotic cells. In contrast, PS exposure was enhanced in cells treated with lethal doses of FTY720. Consistently, the non-apoptotic exposure of PS in vital monocytes also occurs one to two orders of magnitude below apoptotic cells, and cooperative binding of Ann V is said to require a critical density and/or clustering of PS molecules on dying cells^70^.

Cellular vacuolization was identified as a critical factor for FTY720-induced PS flipping and cell death in AML, as plasma membrane asymmetry and viability could be restored upon the removal of FTY720 or by agents that inhibit vacuolization. Previous reports have shown that sphingosine analogs induce the dilation of late endosomes in a sphingosine kinase 1- and cholesterol-dependent manner^58,59^ and downstream of the PP2A-dependent mislocalization of phosphatidylinositol-3-phosphate 5-kinase (PIKfyve)^62^. Notably, vacuolization is required for cell death by such analogs, and this has been elegantly demonstrated by structural activity studies^61^ as well as using genetic models that suppress vacuole formation^60^ or clearance^58^. In addition to vacuolization, sphingolipid analogs downregulate lipid-raft associated nutrient transporters due to impaired membrane recycling resulting from the PP2A-dependent inactivation of Arf6^46^. Interestingly, PS is enriched in recycling endosomes and is essential for endosomal membrane trafficking in both yeast and mammalian cells, as depletion of endosomal phospholipid flippase ATP9A delays the recycling of transferrin and glucose transporter 1 from endosomes to the plasma membrane^71–73^. Moreover, PS flipping is required for the recruitment of the membrane fission protein Eps15-homology domain-containing protein1 (EHD1) to endosomal tubules for vesicle formation^73^. As EHD1 interacts with molecules interacting with CasL-Like1 (MICAL-L1) at tubular recycling endosomes^74^ and sphingolipid analogs induce the loss of MICAL-L1 from endosomal tubules following nutrient transporter internalization^46^, PS flipping at the plasma membrane may disrupt the asymmetric transbilayer distribution of PS on recycling endosomes and contribute to the defects in membrane recycling. Future studies are needed to investigate the crosstalk between plasma membrane asymmetry, membrane recycling and cell death.

While inhibition of PP2A by calyculin A rescued nutrient transporter expression at the plasma membrane, Arf6 was still inactivated under these conditions^46^. We observed that co-treatment with calyculin A and FTY720 maintained plasma membrane asymmetry but significantly increased the generation of subcellular sized particles labeled by Ann V. The externalization of PS accompanies the shedding of plasma membrane-derived microvesicles^75^. Furthermore, nucleotide cycling of Arf6 regulates the phosphorylation of myosin II light chain (MLC) by myosin light-chain kinase (MLCK) to allow for fission of tumor-derived microvesicles containing PS on the surface^76^. Interestingly, non-muscle myosin IIA was identified as a PP2A-associated downstream effector protein in FTY720-treated cells^34^ that promotes FTY720-induced membrane blebbing during type II necroptosis^37^. While further investigation is required, we propose that PP2A activation by FTY720 may suppress MLC/myosin IIA-mediated plasma membrane shedding and repair to promote PS externalization, defective membrane trafficking and cell death.

Interestingly, PS exposure in AML cells was induced by FTY720 but not by the metabolite FTY720-phosphate or fluorescent analog NBD-FTY720. The loss of phenotype upon the addition of a hydrophilic group to the “head” or “tail” of FTY720 suggests that membrane partitioning and/or diffusion may be important for PS flipping. Notably, initial Ann V binding was restricted to 7-AAD-negative cells to indicate that the sphingosine analog was not directly altering plasma membrane integrity or causing membrane dissolution. However, changes in plasma membrane organization have been shown to generate energetically favorable sites for spontaneous PS flopping in a translocase-independent mechanism^77^. In addition, changes in the sphingolipid composition of the plasma membrane can promote translocase-dependent lipid flip-flop, as ceramide domains activate lipid scramblase for flip-flop during red blood cell lysis^78^. As FTY720 targets several enzymes in the sphingolipid pathway, future studies are warranted to dissect whether FTY720 itself or downstream changes in sphingolipid composition regulate PS flipping and cell death in AML cells.

## Supplementary information

Supplementary Figure Legends

Figure S1

Figure S2

Figure S3

Figure S4

Figure S5

Declaration of Contributions to Article

Reporting Checklist
