# Supplementary material for: FTY720 induces non-canonical phosphatidylserine externalization and cell death in acute myeloid leukemia
Source: Cell Death Dis. 2019 Nov 7;10(11):847. doi: 10.1038/s41419-019-2080-5 (PMC6838108; doi:10.1038/s41419-019-2080-5)
Supplement: Supplementary file 1 — Supplementary Figure Legends [file 41419_2019_2080_MOESM1_ESM.docx]

**Supplementary Figure Legends**

**Fig. S1. FTY720 induces PS exposure and dose-dependent cell death in multiple AML cell lines. (A).** MV4-11 cells were treated with DMSO or 7.5 µM FTY720 (2 h or 24 h) or 100 nM ABT-199 (*positive control*, 24 h), stained with FITC-Ann V and 7-AAD and analyzed by flow cytometry. Representative FITC-Ann V histograms are shown. **(B-E).** THP1 (**B**, **C**) or MOLM13 (**D**, **E**) cells were treated with the indicated concentrations of FTY720 in the presence of FITC-Ann V and YOYO3 and imaged using the IncuCyte Live-Cell Analysis System. Image quantification was performed with the Cell-by-Cell Analysis Module. Mean ± SD, n=3. Note: error bars are included for all data points but may be masked by symbols. **B** and **D**, Percent of FITC-Ann V positive cells. **C** and **E**, Percent of FITC-Ann V and YOYO3 double-positive cells. **(F-G).** MV4-11 cells were pre-treated with DMSO or FTY720 (5.0 or 7.5 µM) for 4 h prior to the addition of FITC-Ann V and YOYO3 and imaging by IncuCyte. For wash-out conditions, cells were pretreated as described for 4 h, washed once with medium, and imaged without treatment in the presence of FITC-Ann V and YOYO3. Mean ± SD, n=3. Note: error bars are shown for all data points but may be masked by symbols. **F**, Percent of FITC-Ann V positive cells versus time. **G**, Percent of FITC-Ann V and YOYO3 double-positive cells versus time. **(H).** MV4-11 cells were treated with the indicated doses of FTY720-phosphate (FTY720-P) or FTY720 for 24 h, stained with APC-Ann V and 7-AAD and analyzed by flow cytometry. Mean ± SD, n=3. Statistical significance for the AnnV+7AAD− and AnnV+7AAD+ populations were determined by two-way ANOVA followed by Tukey’s multiple comparison test. ns, not significant; ****, p ≤0.0001. ###, p≤0.001.

**Fig. S2. FTY720-induced effects in AML are independent of apoptosis and necroptosis. (A-B).** THP1 cells were treated with 12.5 μM FTY720 in the presence of absence of 50 µM z-VAD-fmk, 15 µM GSK’872, 1 µM NSA or 30 µM Nec-1 in medium containing FITC-Ann V-AF594 and YOYO3. Images were obtained using the IncuCyte Live Cell Analysis System and quantified using the Cell-by-Cell Analysis module. Mean ± SD, n=3**.** Note: error bars are included for all data but may be smaller than the symbols. **A**, Percent of FITC-Ann V positive cells versus time. **B**, Percent of Ann V- and YOYO3- double-positive cells versus time. **(C).** MOLM13 cells were treated with DMSO or 12.5 µM FTY720 in the presence of absence of 50 µM z-VAD-fmk, 30 µM Nec-1 or 15 µM GSK’872 in medium containing YOYO3. IncuCyte images were quantified using the Cell-by-Cell Analysis module. Mean ± SD, n=3. **(D).** MV4-11 cells were seeded in medium containing CellEvent Caspase-3/7 Green and treated with 7.5 μM FTY720 or 600 nM BV6 plus 20 μM z-VAD-fmk (BV6/zVAD; *positive control*) ± 15 µM GSK’872 or 1 µM NSA for 24 h. Cells were stained with APC-Ann V and 7-AAD and analyzed by flow cytometry. Mean ± SD, n=3. Statistical significance for AnnV+Casp3/7− (*) and AnnV+Casp3/7+ (#) populations was determined by two-way ANOVA followed by Tukey’s multiple comparison test. ns, not significant; ****, p ≤0.0001; ***, p ≤0.001; **, p ≤0.01; ####, p ≤0.0001. **(E).** THP1 were co-treated with 30 ng/mL TNFα, 5 µM BV6 and 25 µM z-VAD-fmk (TBZ; *positive control* for necroptosis) in the presence or absence of 30 µM Nec-1, 15 µM GSK’872 or 1 µM NSA in medium containing FITC-Ann V and YOYO3. IncuCyte images were quantified using the Cell-by-Cell Analysis module. Mean ± SD, n=3. Solid and dashed lines represent Ann V-positive and Ann V/YOYO3 double-positive populations, respectively.

**Fig. S3. FTY720-induced effects in AML are independent of ferroptosis, ROS, and autophagy. (A-B).** THP1 cells were treated with 12.5 μM FTY720 in the presence or absence of 1 μM Fer-1, 5 mM NAC, or 10 µg/mL E64d plus 10 µg/mL pepstatin A (E64d/PepA) in medium containing FITC-Ann V and YOYO3. Images were obtained using the IncuCyte Live-Cell Analysis System and quantified with the Cell-by-Cell Analysis Module. Mean ± SD, n=3. Note: error bars are shown for all treatments but may be smaller than symbols. **A**, Percent of FITC-Ann V positive cells versus time. **B**, Percent of FITC-Ann V and YOYO3 double-positive cells versus time. **(C).** THP1 cells were treated with 5 µM RSL-3 (*positive control* for ferroptosis) or 10 mM hydrogen peroxide (H2O2; *positive control* for ROS) in the presence or absence of 2 µM Fer-1 or 5 mM NAC, respectively, in medium containing FITC-Ann V and YOYO3. IncuCyte images were quantified using the Cell-by-Cell Analysis module. Mean ± SD, n=3. Solid and dashed lines represent Ann V-positive and Ann V/YOYO3 double-positive populations, respectively. **(D).** MOLM13 cells were treated with DMSO or 12.5 µM FTY720 in the presence or absence of 2 µM Fer-1, 5 mM NAC, or E64d/PepA (10 µg/mL each) in medium containing YOYO3. IncuCyte images were quantified with the Cell-by-Cell Analysis Module. Mean ± SD, n=3. **(E-F).** Immunoblots of CRISPR-non-targeting (NT) or CRISPR-ATG7 MOLM13 (**E**) or THP1 (**F**) cell lines probed with the indicated antibodies. **(G).** CR-NT or ATG7-deficient MOLM13 cells were treated with 10 µM FTY720 for 24 h, stained with APC-Ann V and 7-AAD and analyzed by flow cytometry. Mean ± SD, n=3. Statistical significance for AnnV+7AAD− (*) and AnnV+7AAD+ (#) populations was determined by two-way ANOVA followed by Tukey’s multiple comparison test. ns, not significant; ****, p ≤0.0001. **(H).** CR-NT or ATG7-deficient THP1 cells were treated with 14 µM FTY720 for 24 h, stained with APC-AnnV and 7-AAD and analyzed by flow cytometry. Mean ± SD, n=3. Statistical significance for AnnV+Casp3/7− (*) and AnnV+Casp3/7+ (#) populations was determined by two-way ANOVA followed by Tukey’s multiple comparison test. ns, not significant; *, p ≤0.05; ####, p ≤0.0001.

**Fig. S4. NBD-FTY720 fails to mimic FTY720 and FTY720-induced PS externalization occurs in a cholesterol-dependent manner. (A).** MV4-11 cells were treated with DMSO, FTY720 (3.25 µM or 7.50 µM) or NBD-FTY720 (3.25 µM or 7.50 µM) for 4 h, stained with APC-Ann V and 7AAD and analyzed by flow cytometry. Representative APC-Ann V histograms are shown. **(B-C).** THP1 cells were treated with DMSO or 13 µM FTY720 in the presence or absence of 2 mM methyl-β-cyclodextrin (mβCD) for 4 h (**B**) or 24 h (**C**), stained with APC-Ann V and 7AAD and analyzed by flow cytometry. Mean ± SD, n = 3. Statistical significance for AnnV+7AAD− (*) and AnnV+7AAD+ (#) populations was determined by two-way ANOVA followed by Tukey’s multiple comparison test. ns, not significant; ****, p ≤0.0001; **, p ≤0.01; ####, p ≤0.0001. **(D).** MOLM13 cells were treated with a dose-response of FTY720 in the presence or absence of 2 mM mβCD in medium containing FITC-Ann V and YOYO3. Cells were imaged using the IncuCyte Live Cell Analysis system and quantified with the Cell-by-Cell Module. Solid lines/filled symbols and dashed lines/open symbols represent the absence or presence of mβCD, respectively. Mean ± SD, n = 3.

**Fig. S5. FTY720-induced CD98 internalization and PP2A inhibition by calyculin A promotes the shedding of Ann V-positive subcellular sized particles. (A).** MV4-11 cells were treated with DMSO or 7.5 µM FTY720 for 4 h, stained with unconjugated anti-CD98 Ab and APC-conjugated goat anti-mouse Ig secondary Ab (anti-CD98), IgG1κ isotype control Ab and APC-conjugated goat anti-mouse Ig secondary Ab (Isotype Ctrl), or APC-conjugated goat anti-mouse Ig secondrary Ab alone (2° only Ctrl), and analyzed by flow cytometry. Representative APC histograms of viable cells. **(B-D).** MV4-11 cells were pre-treated with 5 nM calyculin A (CalA) for 30 min followed by the addition of DMSO or 7.5 µM FTY720 for 4 h. (**B**) Representative forward scatter (FSC) and side scatter (SSC) flow cytometry plot indicating the gating of cells (“Cells”) and subcellular sized particles (“P”). (**C**) Representative APC-Ann V/7AAD flow cytometry plot of cells from **B**. (**D**), Representative APC-Ann V/7AAD flow cytometry plot of subcellular sized particles “P” from **B**.
