# Supplementary figures and images for: FTY720 induces non-canonical phosphatidylserine externalization and cell death in acute myeloid leukemia
Source: Cell Death Dis. 2019 Nov 7;10(11):847. doi: 10.1038/s41419-019-2080-5 (PMC6838108; doi:10.1038/s41419-019-2080-5)

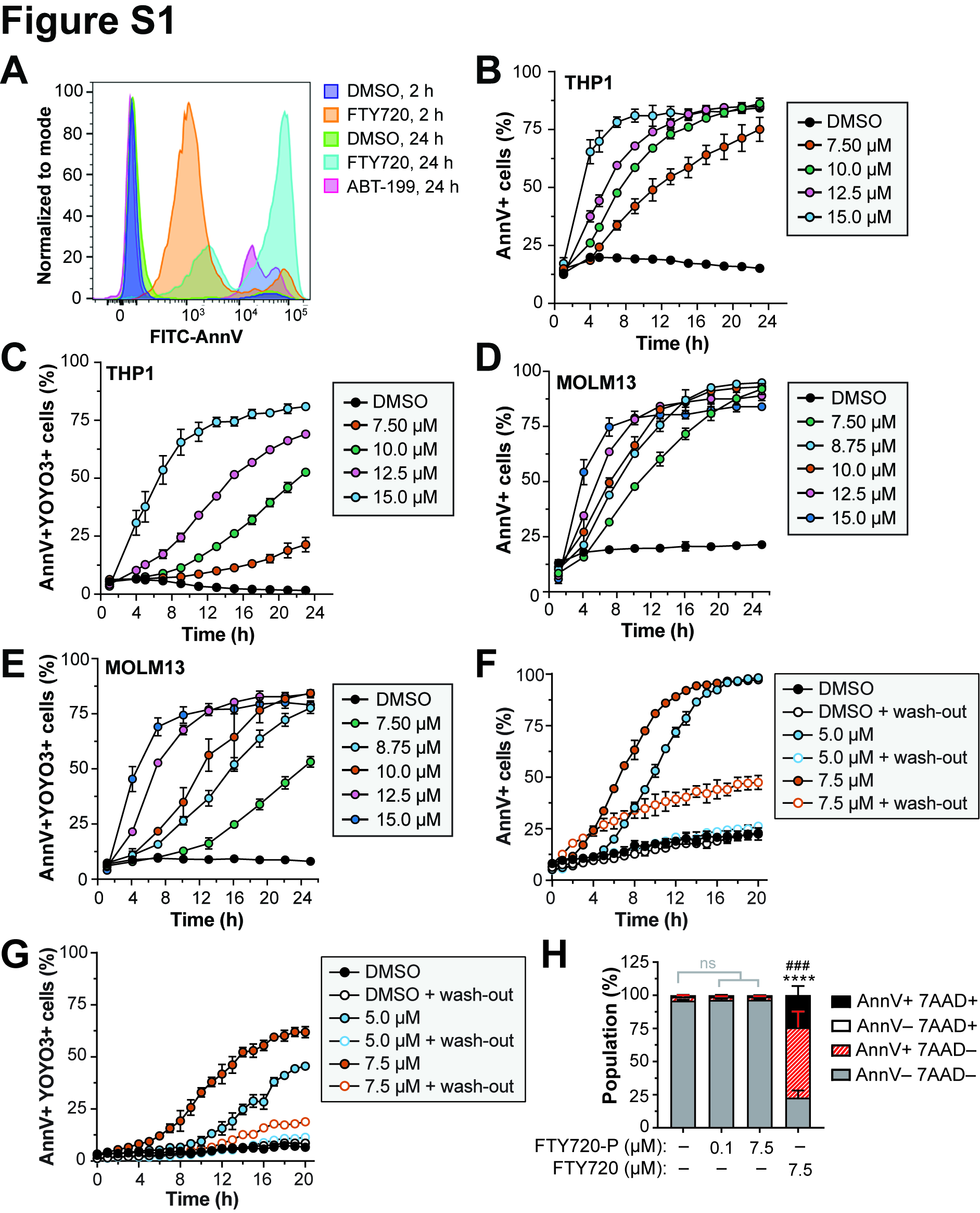

Supplement: Supplementary file 2 — Figure S1 [file 41419_2019_2080_MOESM2_ESM.tif]

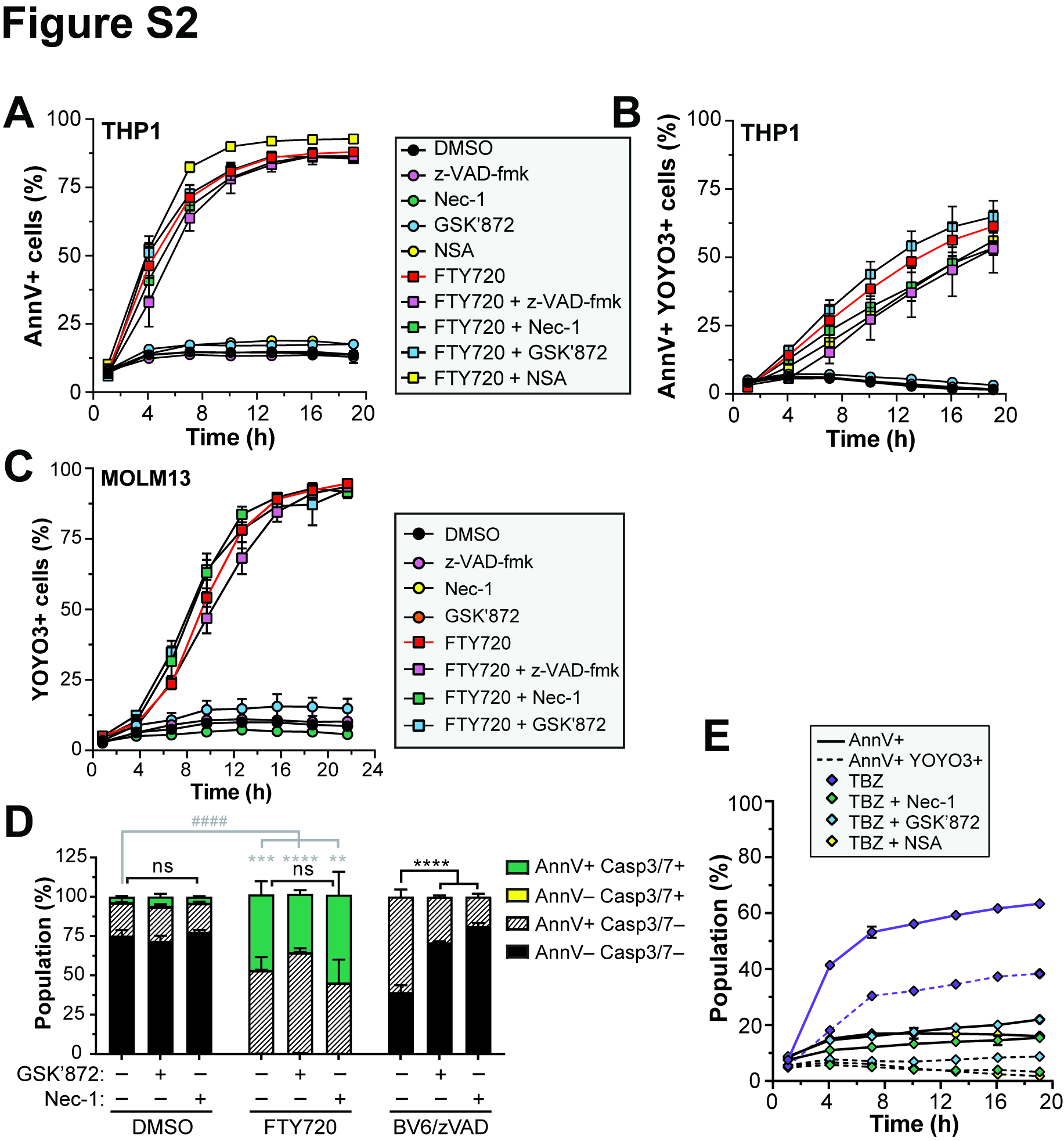

Supplement: Supplementary file 3 — Figure S2 [file 41419_2019_2080_MOESM3_ESM.tif]

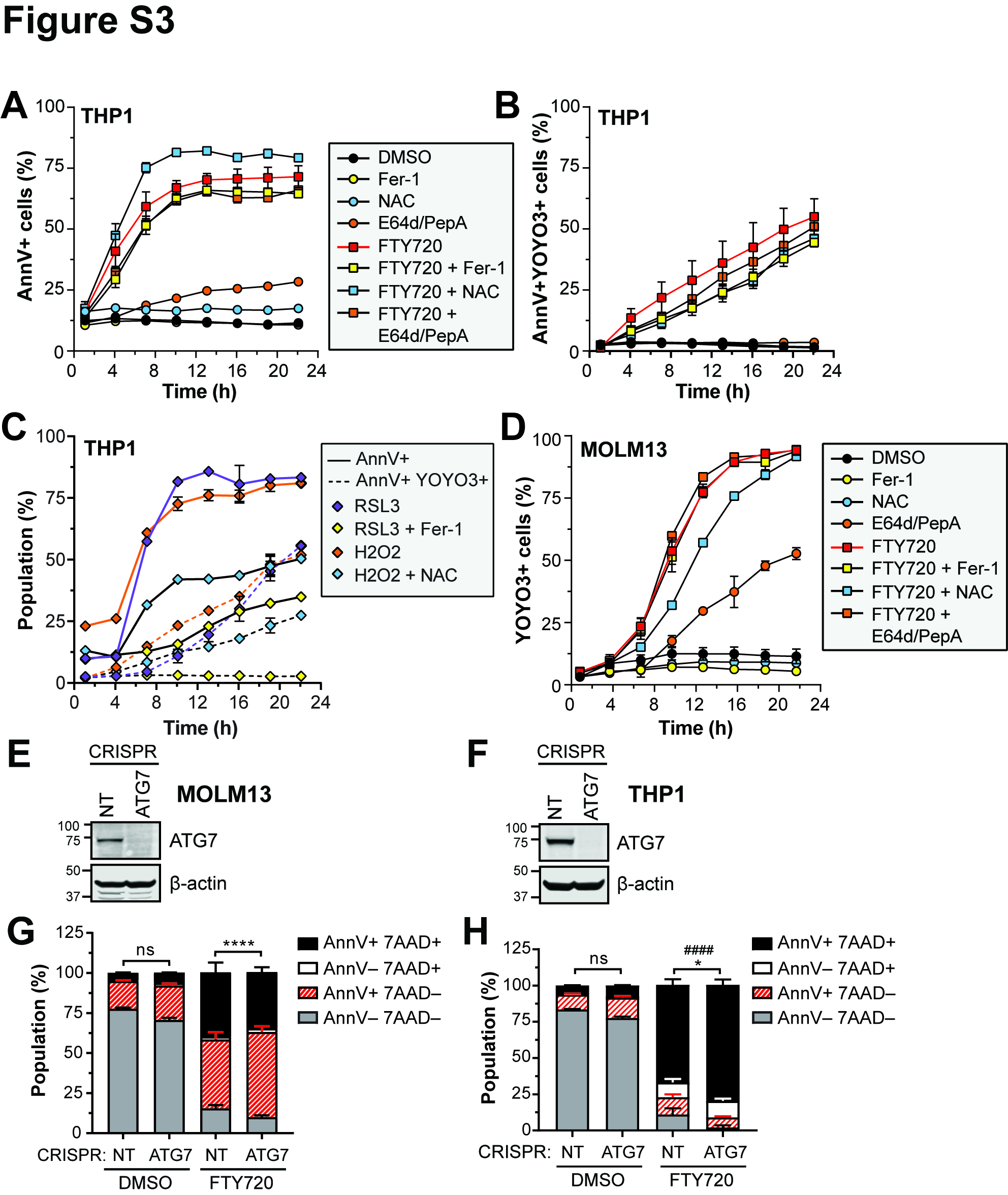

Supplement: Supplementary file 4 — Figure S3 [file 41419_2019_2080_MOESM4_ESM.tif]

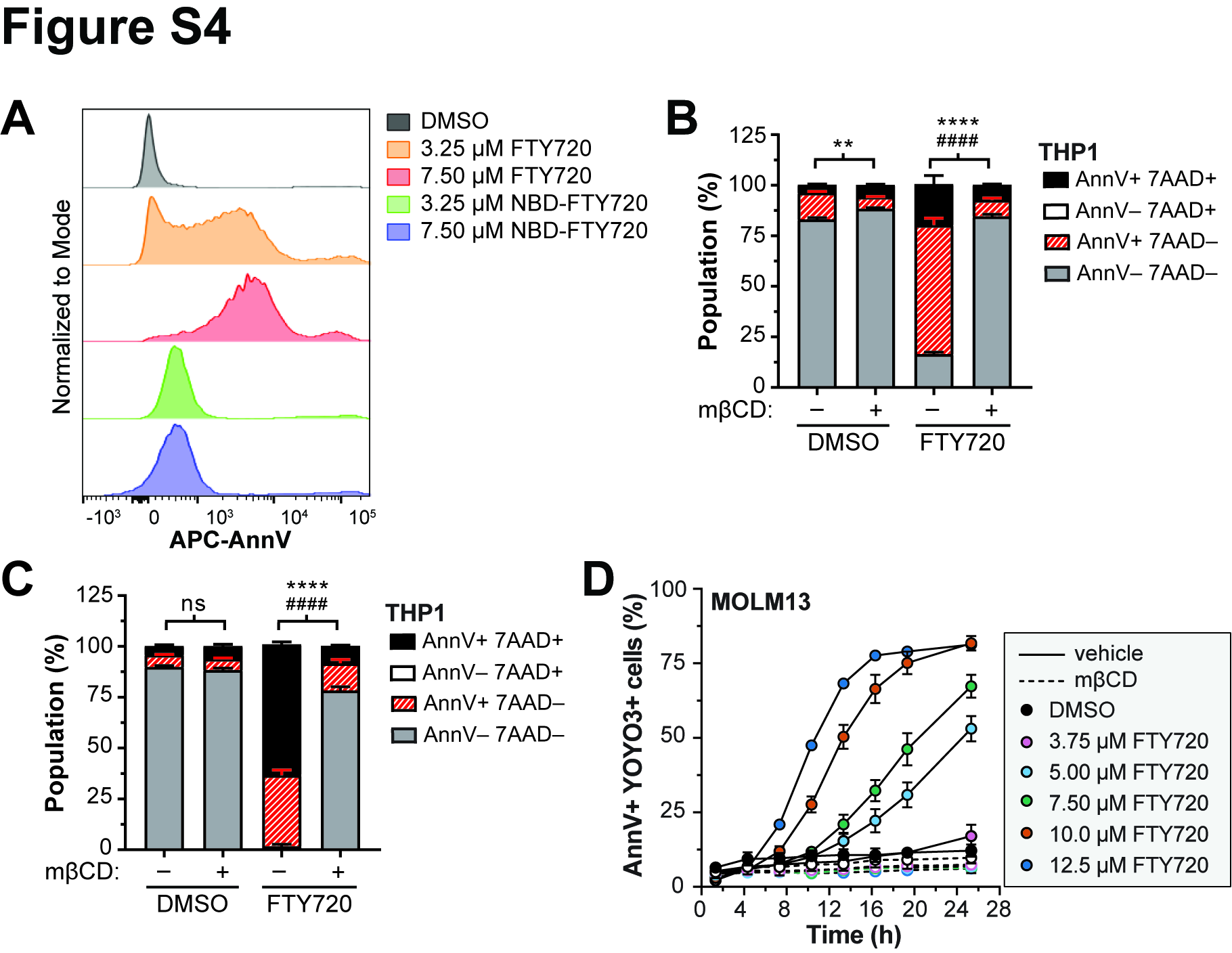

Supplement: Supplementary file 5 — Figure S4 [file 41419_2019_2080_MOESM5_ESM.tif]

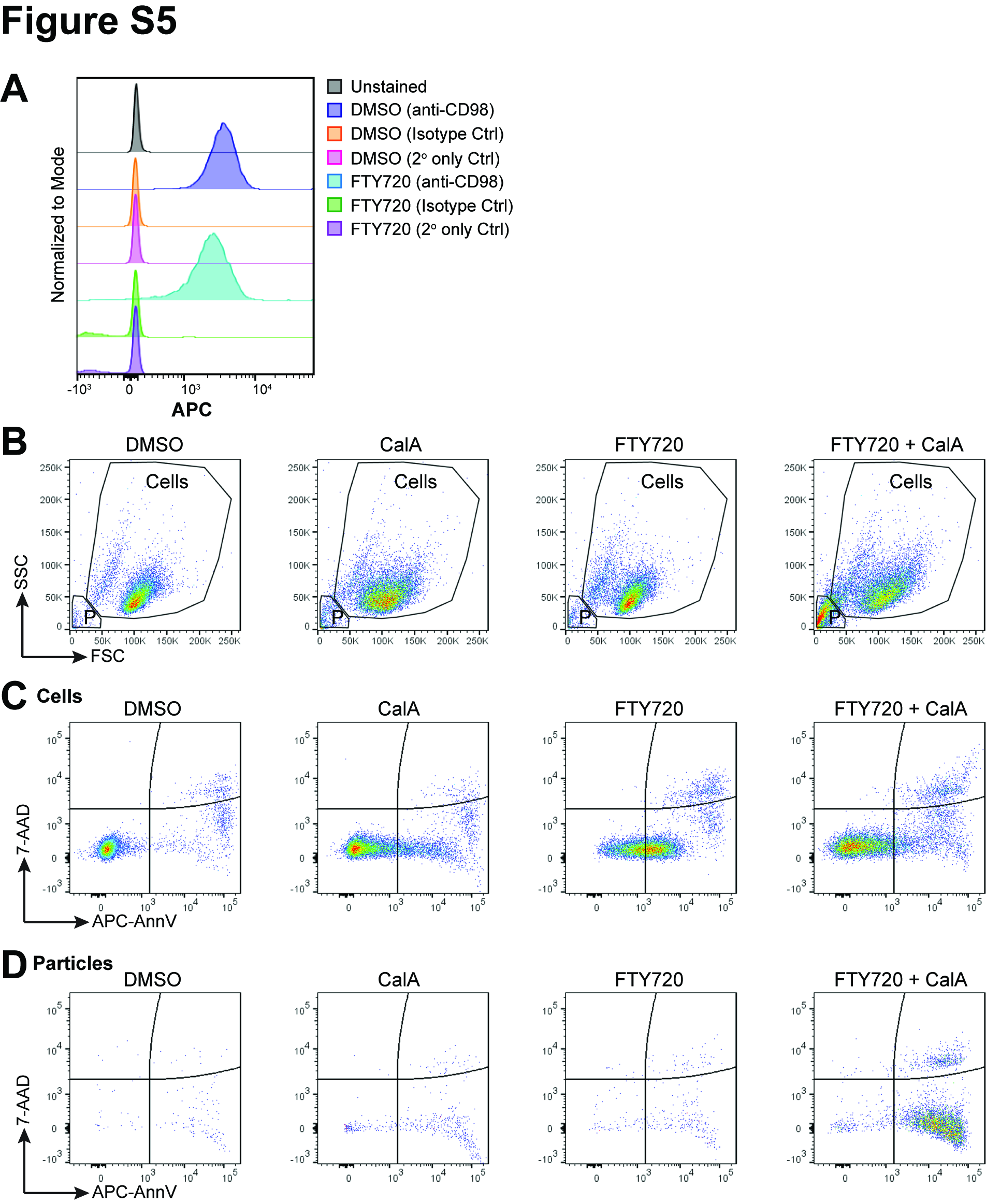

Supplement: Supplementary file 6 — Figure S5 [file 41419_2019_2080_MOESM6_ESM.tif]
