# Supplementary material for: FTY720 induces non-canonical phosphatidylserine externalization and cell death in acute myeloid leukemia
Source: Cell Death Dis. 2019 Nov 7;10(11):847. doi: 10.1038/s41419-019-2080-5 (PMC6838108; doi:10.1038/s41419-019-2080-5)
Supplement: Supplementary file 7 — Declaration of Contributions to Article [file 41419_2019_2080_MOESM7_ESM.pdf]

DECLARATION OF CONTRIBUTIONS TO ARTICLE

ADMC

Manuscript Number:	Journal Name:	
CDDIS-19-3051	*Cell Death & Disease*	(the 'Journal')

Proposed Title of the Contribution:

FTY720 induces non-canonical phosphatidylserine externalization and cell death in acute myeloid leukemia (the 'Contribution')

Author(s):

Megan M. Young, Van Bui, Chong Chen, Hong-Gang Wang (the 'Authors')

For all *CDDis* articles, each person named as an author in the published version must be able to show he or she has contributed substantially to the article.

Authorship credit should be based on 1) substantial contributions to conception and design, acquisition of data, or analysis and interpretation of data; 2) drafting the article or revising it critically for important intellectual content; and 3) final approval of the version to be published. Authors should meet conditions 1, 2 and 3.

Any person who cannot be shown to have made a substantial contribution to the article cannot be listed as an author in the final version. The name of any person who is deemed to have made a minor contribution can, however, appear in the Acknowledgments section of the article.

Please complete the table below to indicate the contributions of all named authors to the manuscript.

Author Full Name:	Specification of Contribution to the Manuscript:
Megan M. Young	MMY conceptualized and performed the experiments, analyzed and interpreted the data, drafted the manuscript and figures, performed revisions to the manuscript, and gave final approval of the version to be published.
Van Bui	VB designed and performed experiments, interpreted the data, provided critical feedback and gave final approval of the version to be published.
Chong Chen	CC designed, established and validated important genetically modified cell lines used in the experiments, interpreted the results, and gave final approval of the version to be published.
Hong-Gang Wang	HGW provided critical feedback on the conceptualization and interpretation of the data, provided resources and funding, critically reviewed the article and figures, revised the manuscript, and gave final approval of the version to be published.

Please complete the table below to indicate the contributions of all named authors to the figures.

Figure 1:

MMY generated the data in all panels and prepared the figure.

Figure 2:

MMY generated the data in all panels and prepared the figure.

Figure 3:

MMY generated the data in Panels A, C, D and E and prepared the figure.
VB generated the data in Panel B and F, and assisted in collecting data in Panels C and D.
CC designed, generated and validated knock-out cell lines in Panel E.

Figure 4:

MMY generated the data in all panels and prepared the figure.

Figure 5:

MMY generated the data in all panels and prepared the figure.

Figure 6:

Signed for and on behalf of the Author(s):	Print Name:	Date:
[signature]	Megan M Young	10/16/2019
